# Gain-Type Aneuploidies Influence the Burden of Selective Long Non-Coding Transcripts in Colorectal Cancer

**DOI:** 10.3390/ijms25105538

**Published:** 2024-05-19

**Authors:** Chiara Scuderi, Virginia Di Bella, Anna Provvidenza Privitera, Francesca Maria Giustolisi, Vincenza Barresi, Daniele Filippo Condorelli

**Affiliations:** Department of Biomedical and Biotechnological Sciences, University of Catania, 95123 Catania, Italy; chiara.scuderi@phd.unict.it (C.S.); virginia.dibella@phd.unict.it (V.D.B.); anna.privitera@phd.unict.it (A.P.P.); francesca_gi@libero.it (F.M.G.); daniele.condorelli@unict.it (D.F.C.)

**Keywords:** chromosome aberrations, cancer aneuploidy, transcriptome, cancer genomics, colorectal cancer, chromosomal instability, omics technologies, lncRNAs, NORAD, SNHG6

## Abstract

Chromosomal instability is a hallmark of colorectal carcinogenesis and produces an accumulation of different forms of aneuploidies or broad copy number aberrations. Colorectal cancer is characterized by gain-type broad copy number aberrations, specifically in Chr20, Chr8q, Chr13 and Chr7, but their roles and mechanisms in cancer progression are not fully understood. It has been suggested that broad copy number gains might contribute to tumor development through the so-called caricature transcriptomic effect. We intend to investigate the impact of broad copy number gains on long non-coding RNAs’ expression in colorectal cancer, given their well-known role in oncogenesis. The influence of such chromosomal aberrations on lncRNAs’ transcriptome profile was investigated by SNP and transcriptome arrays in our series of colorectal cancer samples and cell lines. The correlation between aneuploidies and transcriptomic profiles led us to obtain a class of Over-UpT lncRNAs, which are transcripts upregulated in CRC and further overexpressed in colon tumors bearing specific chromosomal aberrations. The identified lncRNAs can contribute to a wide interaction network to establish the cancer driving effect of gain-type aneuploidies.

## 1. Introduction

### 1.1. Genomic Aberrations in Colorectal Cancer

The underlying mechanisms responsible for the onset and development of colorectal cancer (CRC) include a variety of genetic and epigenetic changes in colonic epithelial cells, such as chromosomal instability (CIN), microsatellite instability (MSI), CpG island methylator phenotype (CIMP) and high single nucleotide mutation rates (hypermutation-ultramutation) [1,2]. CIN, detected in about 80–85% of CRC, is characterized by alterations in chromosomal number and structure, including aneuploidy, somatic copy number alterations (SCNAs) and copy-neutral loss of heterozygosity (CN-LOH) [3,4]. Aneuploidy refers to an abnormal number of chromosomes in a cell, a condition that typically arises due to errors in cell division, particularly when chromosomes fail to separate correctly, which is known as nondisjunction. However, in cancer research, the definition of aneuploidy has been extended from the original one, based on chromosome numerical abnormalities, to a structural one that also includes copy number abnormalities due to gain or loss of a large portion of a chromosomal arm, or an entire chromosome [5]. CN-LOH refers to a frequent genetic aberration, involving loss of a single copy of a DNA region compensated by duplication of the remaining copy, resulting in no change in gene copy number [6]. The role of CN-LOH is particularly significant in carcinogenesis, since it may contribute to the inactivation of tumor suppressor genes converting monoallelic into biallelic mutations [7]. The copy number alterations (CNAs), gains or losses, can be “Focal” if they affect less than 20–25% of the chromosomal arm, or they can be “Broad” if they affect a larger part of the chromosome, up to involving entire chromosomal arms or even entire chromosomes [4,8,9]. Therefore, the presence of broad copy number aberrations (BCNAs) can be considered an alternative definition for aneuploidy. Indeed, an aneuploidy index has been devised based on the counting of arm-level chromosomal gains and losses [5].

### 1.2. LncRNAs: Classification, Function and Involvement in Cancer

Long non-coding RNAs (lncRNAs) are defined as non-coding transcripts longer than 200 nucleotides [10]. This ever-increasing class of transcripts is highly heterogeneous due to the wide variety of functions, mechanisms of action and genomic localization that characterize them. LncRNAs can be transcribed from coding or non-coding regions, including the introns or intergenic sequences. Some coding genes can generate transcripts encoding proteins and, in addition, non-coding transcripts having regulatory functions independent of their translation. These protein-coding transcripts are classified as “coding/non-coding RNAs” (cncRNAs) [11,12]. Moreover, the transcribed lncRNAs, derived from protein-coding genes, can be sense- or antisense-overlapping. In other cases, non-coding transcripts can derive from intronic and intergenic regions and are called intronic lncRNAs and intergenic lncRNAs (lincRNAs), respectively. The latter (lincRNAs) are defined as RNA transcripts that are longer than 200 nucleotides and do not overlap protein-coding genes. Otherwise, lncRNAs can be transcribed from enhancer regions or they are circular RNAs (CircRNAs) that originated through back-splicing of coding and non-coding transcripts [10,13]. Furthermore, lncRNAs can be found in different subcellular compartments, including the nucleus, cytoplasm, mitochondria or exosomes. According to their subcellular localization, lncRNAs can be transcribed in sense and antisense directions and regulate gene expression at multiple levels, contributing to chromatin, transcriptional and post-transcriptional regulation by lncRNA-DNA, lncRNA-RNA or lncRNA-protein interactions [10]. Cis-acting and trans-acting nuclear lncRNAs can modulate chromatin structure by establishing direct or indirect interactions with DNA. Direct interactions between lncRNAs and DNA involve the formation of hybrid structures, such as triplexes or loops, which influence chromatin accessibility. Alternatively, lncRNAs can interact with chromatin remodeling complexes and recruit them to target DNA regions. LncRNAs participate in transcriptional regulation as well, by interfering with the recruitment of transcription factors or RNA Polymerase II to the gene target’s promoter. Regarding post-transcriptional regulation, lncRNAs are known to interact with specific RNA-binding proteins, resulting in alteration of mRNA splicing, regulation of mRNA turnover and modulation of signaling pathways. Moreover, it has been reported that some lncRNAs can directly base pair with RNAs and modulate their translation. In addition, gene expression can be regulated through the so-called “Competitive endogenous RNA (ceRNA) network”, based on the interaction between lncRNAs, miRNAs and mRNAs [10,13,14]. In order to repress gene expression, miRNAs bind to partially complementary sequences, known as microRNA response elements (MREs), located on coding and non-coding transcripts. Thus, all types of RNAs, sharing MREs, will crosstalk, generating a large-scale RNA regulatory network. The ceRNA network is the basis of the “sponging” mechanism involved in gene expression. In particular, lncRNAs bearing the same MREs of target mRNAs, will act as “sponges” of miRNAs, reducing their availability and, thus, inhibiting their binding to target mRNAs [15]. 

Comprehensive analysis of transcriptome profiles across different cancer types has revealed that several lncRNAs are characterized by aberrant expression that may consist in either upregulation or downregulation, compared to normal tissues. According to the pathways in which they are involved, lncRNAs may act as oncogenes or as tumor suppressors [10,16,17]. The literature reports several oncogenic lncRNAs associated with tumor proliferation, metastasis and poor prognosis in colorectal cancer, such as PVT1, NORAD, CCAT1, CCAT2 and CRNDE [18,19,20,21]. In contrast, known long non-coding RNAs (lncRNAs) acting as tumor suppressors in colorectal cancer include CASC2 and GAS5 [22,23,24,25]. Thus, dysregulated lncRNAs play a substantial role in the initiation and development of tumors, suggesting their potential utility as diagnostic and prognostic markers for cancer progression [26].

In the present work we aimed to identify candidate lncRNAs that could take part in the transcriptional dysregulation induced by broad copy number gains, a form of aneuploidy. We used a transcriptomic strategy based on the so-called “caricature” effect [8] and focused on gain-type aneuploidy because this type of aberration is likely to act through an increased copy-number-dependent gene transcription. Indeed, in several early and frequent gain-type aneuploidies, such as trisomy or tetrasomy of Chr20 in colon cancer, no alternative cancer-driving mechanism has been revealed apart from a clear-cut transcriptional dysregulation, and due to this, an exploration of the role of lncRNAs is warranted. No increased copy number of genes bearing activating mutations has been clearly shown in this kind of chromosomal aberration. On the contrary, loss-type aneuploidy can represent the second hit for the inactivating point mutations of tumor suppressor genes on the homologous chromosome, making it more difficult to propose a transcriptomic strategy for the identification of cancer driver genes.

## 2. Results

### 2.1. Chromosomal Distribution of BCNAs and Somatic Broad Copy-Neutral Loss of Heterozygosities (SB-CNLOHs) in Colorectal Cancer Cell Lines

BCNAs, as defined by Condorelli et al., are chromosomal aberrations that involve more than 25% of a chromosomal arm (p or q) or a whole chromosome (w), distinguished in broad losses and broad gains [4]. As previously reported, ≥25% of the analyzed CRC samples report a high frequency of broad gains in 20q/w, 8q/w, 13, 7q/w, Xq/w and 9q/w, and a high frequency of broad losses in 18q/w, 8p, 17p and 15q [8].

In this paper, a genome-wide DNA copy number was performed to verify chromosomal distribution of BCNAs in colorectal cancer cell lines. The SNP array analysis allows identification of CNLOH regions. The sum of deleted genes (i.e., copy number −2 and −1), diploid genes (copy number 0) and amplified genes (copy number +1, +2) for all chromosomal arms was calculated for each cancer cell line. 

**Figure 1:**
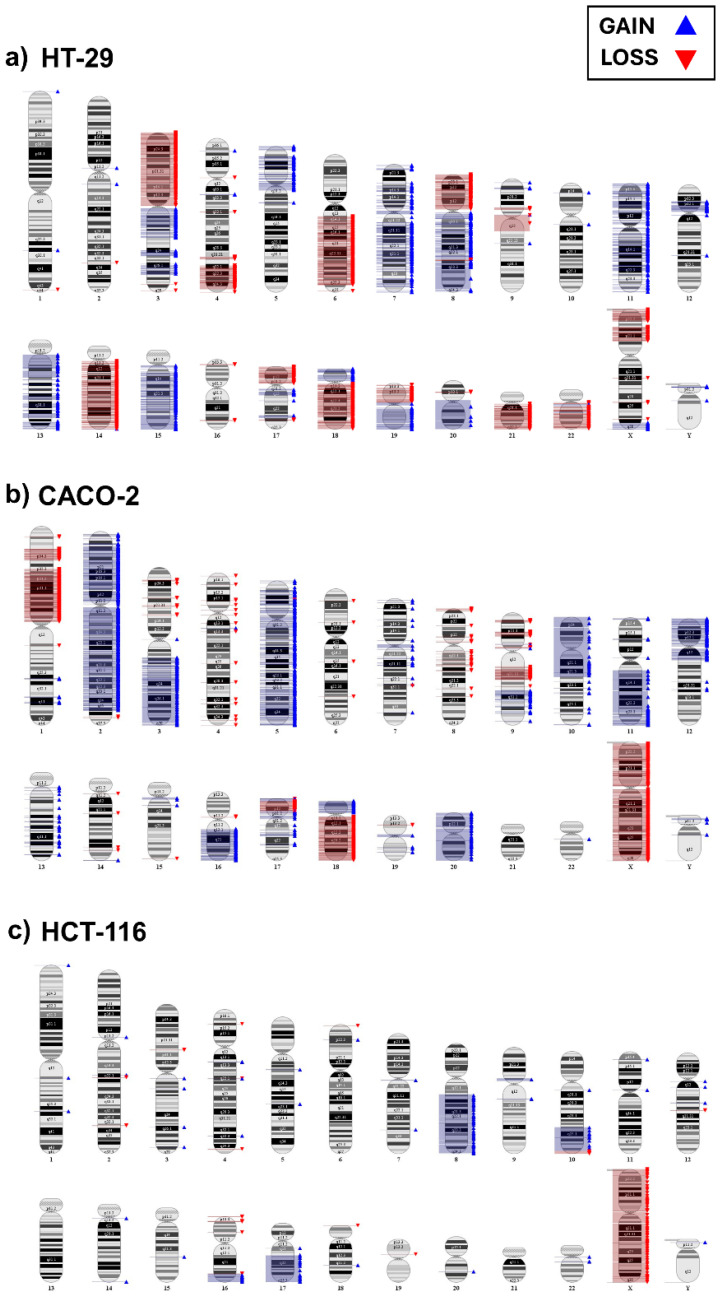
Molecular Karyoview of all chromosomal aberrations obtained by the Affymetrix Genotyping Console in colorectal cancer cell lines. (**a**) HT-29 virtual karyotype; (**b**) CACO-2 virtual karyotype; (**c**) HCT-116 virtual karyotype. Gains and losses are represented on chromosome ideograms by blue and red triangles, respectively.

HT-29 is characterized by several aneuploidies, including several focal and broad aberrations. Among broad aberrations, worthy of note are gains of chromosomes 5p, 7, 8q, 11, 13q, 15q and 19q, and losses of chromosomes 3p, 6q, 8p, 14q, 18q, 21 and 22. The virtual karyotype is shown in Figure 1a.

The CACO-2 cell line is characterized by several aneuploidies, including both focal and broad aberrations. The latter include gains of chromosomes 2, 3q, 5, 12p, 13, 16q, 18p and 20, and losses of chromosomes 1p and 18q (Figure 1b).

The HCT-116 cell line is characterized by a near-diploid karyotype, except for the gain of chromosomes 8q and 17q, as illustrated in Figure 1c.

The SNP array analysis showed the highest frequency of SB-CNLOHs in highly aneuploid colon cancer cell lines CACO-2 and HT-29, while only one CNLOH in Chr3p has been observed in the near-diploid HCT-116.

### 2.2. LncRNAs’ Transcriptional Effects Associated with Gain-Type BCNAs in CRC Samples and Cell Lines

The influence of gain-type BCNAs on lncRNAs’ transcriptome profile was investigated by SNP and transcriptome arrays in our series of **c**olo**r**ectal **c**ancer (CRC) samples and cell lines. CRC samples were organized in four groups, based on the presence of selected gain-type BCNAs or their absence in the corresponding control groups, as follows: all CRC, Control CRC, Selected CRC and the Normal Colonic Mucosae group. CRCs with a specific gain aberration form the “Selected CRC” group, while CRC without any chromosomal aberration on the chosen chromosome form the “Control CRC” group [8]. 

Transcriptional effects associated with gain-type BCNAs could identify lncRNAs that are differentially expressed (at the transcript level) between the CRC group and corresponding normal colonic tissues (so-called UpT lncRNAs), or between the Selected CRC group and the corresponding Control CRC group (so-called OverT lncRNAs).

Fold-changes (FC) were obtained through a comparison of average RMA values between the different groups. Four different indices were used in order to estimate transcript levels in CRC and to define the following different transcript classes: linear fold-changes obtained by comparing all CRC samples to matched Normal Colonic Mucosae (denominated FC1), linear fold-changes obtained by comparing CRCs bearing a specific BCNA (Selected CRC group) to CRCs not bearing a specific BCNA (Control CRC group) (denominated FC2), linear fold-changes obtained by comparing the Control CRC group to Normal Colonic Mucosae (denominated FC3) and linear fold-changes obtained by comparing the Selected CRC group to Normal Colonic Mucosae (denominated FC4) [8].

#### 2.2.1. List of UpT LncRNAs

In order to obtain the list of “Upregulated Transcripts” (UpT) between all of the CRC groups and corresponding normal colonic tissues the following fold changes cut-off are adopted: FC3 and FC4 > 1.5. A list of 55 “UpT lncRNAs”, transcripts whose expression increases in CRC compared to mucosae, is reported in Table 1 for selected chromosome gains, shown according to their chromosomal location from p telomere to q telomere, as follows: 22 located in Chr7, 9 in Chr8q, 7 in Chr13 and 17 in Chr20. The analysis has been specifically conducted on annotated lncRNAs that have a designed transcript cluster on HTA 2.0. The NR_accession number and fold-change values are reported in Table 1 and Table S1.

#### 2.2.2. List of Over-UpT LncRNAs in CRC

**Figure 2:**
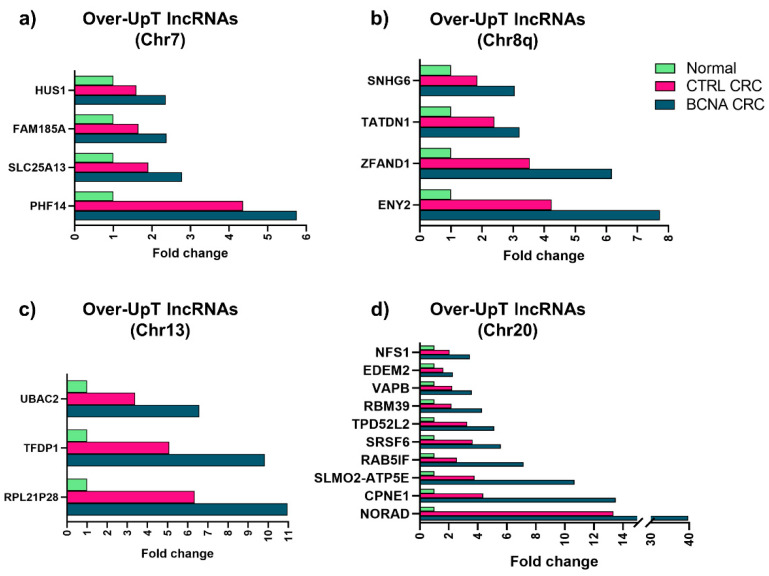
Fold-changes of representative “Over-UpT” lncRNAs located in Chr7, Chr8q, Chr13 and Chr20. The plots show transcript levels in Normal Colonic Tissue (Normal), in the Control (CTRL) CRC group and in the Selected CRC group (BCNA CRC). Transcript levels are expressed as fold-change relative to values in normal tissue (FC3 or FC4).

In order to generate a list of Over-UpT lncRNAs located in gain-type BCNA regions, we adopted the combination of two cut-off values, FC2 > 1.3 and FC3 > 1.5, thus obtaining the list of the so-called Over-UpT lncRNAs that are upregulated in cancer and further overexpressed in tumors bearing a specific broad copy number gain compared to the Control CRC group that does not carry the aberration. Therefore, such Over-UpTs are transcribed from genes which might play a general role in cancer initiation and progression, and are further potentiated by a copy-number-dependent mechanism, underlying the so-called “caricature transcriptome effect” [8]. The use of the above-mentioned filters produced a subset of 21 “Over-UpT” lncRNAs, demonstrating the positive influence of copy number gains on lncRNAs’ expression. We have identified four lncRNAs in Chr7, six in Chr8q, three in Chr13 and ten in Chr20. Increased fold-change values of representative Over-UpT lncRNAs in Chr7, Chr8q, Chr13 and Chr20 are reported in Figure 2. 

#### 2.2.3. Confirmation of lincRNAs and Small Nucleolar Host Gene Transcripts as Over-UpT Using RNA-seq Data from The Cancer Genome Atlas (TGCA) Study

In order to compare data from our local cohort of CRC samples analyzed using HTA 2.0 to a larger group of cancer samples analyzed using a different omics technique (RNA-sequencing), we downloaded data on TCGA colon adenocarcinoma (COAD) samples from The Genomic Data Commons (GDC) Data Portal (https://portal.gdc.cancer.gov accessed on December 2019) [27,28].

Primary COAD samples (n = 433) and 41 colon mucosal normal samples, having both RNA-seq data and cytogenetic results (Affymetrix SNP 6.0 arrays), have been analyzed for RNA expression. Differential expression of transcripts used the same strategy previously described for the local CRC cohort. We organized TCGA-COAD samples in groups bearing a specific chromosomal aberration (selected COAD group: Chr20-gain, Chr8q- gain, Chr13-gain and Chr7-gain). Each “Selected COAD group” was compared with a corresponding “Control COAD” group composed of tumors lacking any broad CNAs on the chosen chromosome.

**Figure 3:**
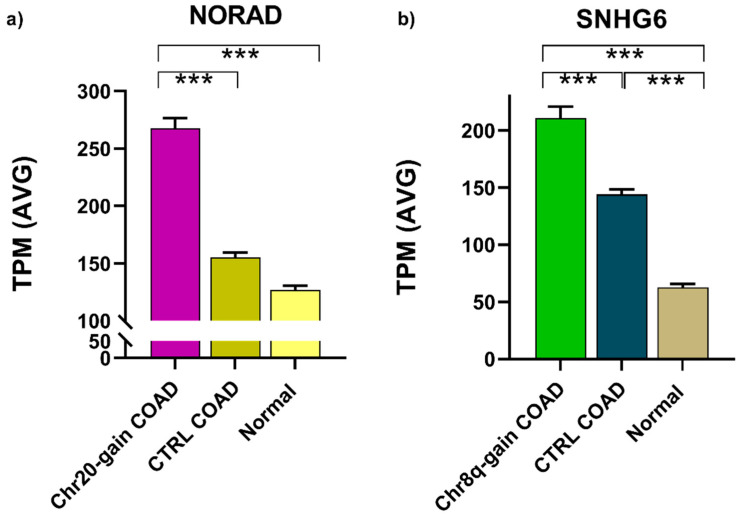
TPM values of NORAD in Chr20-gain-type (**a**) and SNHG6 in Chr8q-gain-type (**b**) are reported for each group analyzed (mean and SEM) (*** *p*-value < 0.001).

Since RNA-sequencing data provided by the TCGA study do not distinguish between coding and non-coding transcripts of protein-coding genes or among transcripts derived by coding genes or their pseudogenes, we focused on lincRNAs and small nucleolar host gene RNAs. Only one of the Over-UpT lncRNAs identified in microarray experiments on Chr20 is a lincRNA (NORAD), the other transcripts belonging to the protein-coding gene sense-overlapping class. Analysis of the expression level of NORAD in COAD samples of the TCGA study (Figure 3a) confirmed its pattern as Over-UpT lncRNA. Among Over-UpT lncRNAs identified on Chr8q in microarray experiments, only SNHG6 is a small nucleolar host gene transcript, while the remaining transcripts were protein-coding sense-overlapping ones. Furthermore, SNHG6 confirmed its behavior as an Over-UpT transcript in the TGCA study (Figure 3b). 

### 2.3. Expression of NORAD lncRNA in CRC Samples and Cell Lines

**Figure 4:**
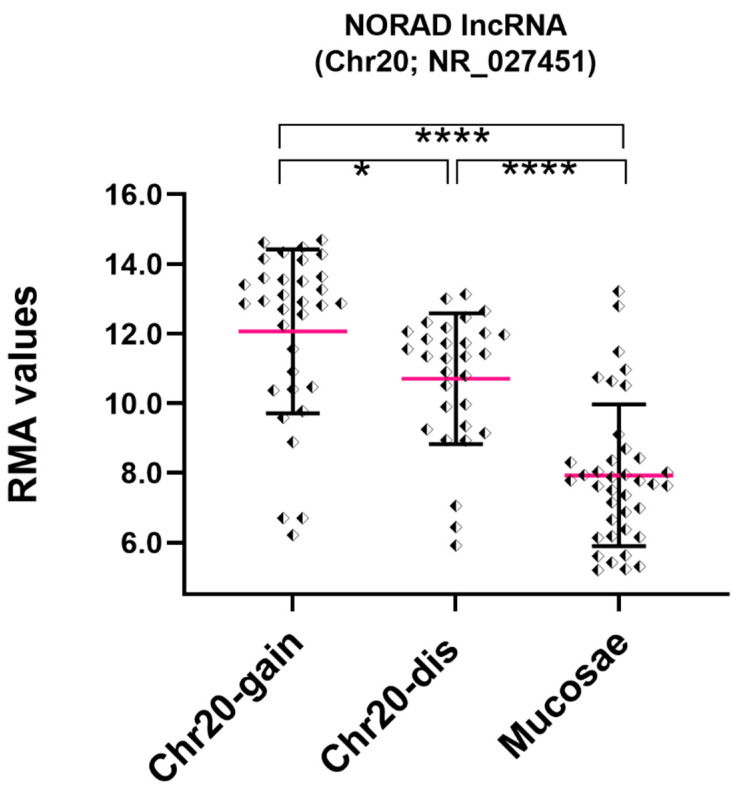
RMA values of NORAD in CRC (Chromosome 20 gain and disomic) and mucosae samples (FC3 > 1.5, FC2 > 1.3) obtained by HTA 2.0. Fuchsia line represents mean value (mean and SD) (* *p*-value < 0.05; **** *p*-value < 0.0001).

Among Over-UpT lncRNAs confirmed in our cohort and in the TCGA study, worthy of note is NORAD lncRNA (NR_027451; 5,343 bp; 1 exon), a “non-coding RNA activated by DNA damage”, located on Chr20. It is characterized by having a higher expression in cancer samples with disomic Chr20 in comparison to mucosae (UpT, 12-fold) and an increased expression in samples bearing Chr20-gain versus disomic Chr20 (Over-UpT, 3-fold) (Figure 4).

**Figure 5:**
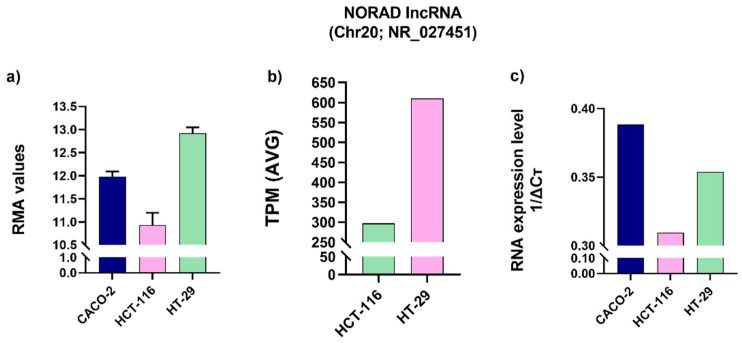
NORAD expression in colorectal cancer cell lines: RMA (**a**)**,** TPM (**b**) values and q-RT PCR (**c**).

The correlation between aneuploidies and the transcriptomic profile for NORAD was also investigated in colon cancer cell lines using HTA and RNA-seq data (Figure 5a,b). Genomic analysis by SNP array 6.0 reported in Section 2.1 showed that HT-29 and CACO-2 cell lines are characterized by Chr20-gain, while the HCT-116 cell line does not carry it. Figure 5a reports the RMA value of NORAD in colon cell lines, confirming the dosage effect of gain-type BCNAs on expression.

Quantitative PCR performed for NORAD in three cancer cell lines confirms a higher level of NORAD in Chr20-gain positive cell lines (CACO-2 and HT29) (Figure 5c). 

### 2.4. Expression of SNHG6 lncRNA in CRC Samples and Cell Lines

**Figure 6:**
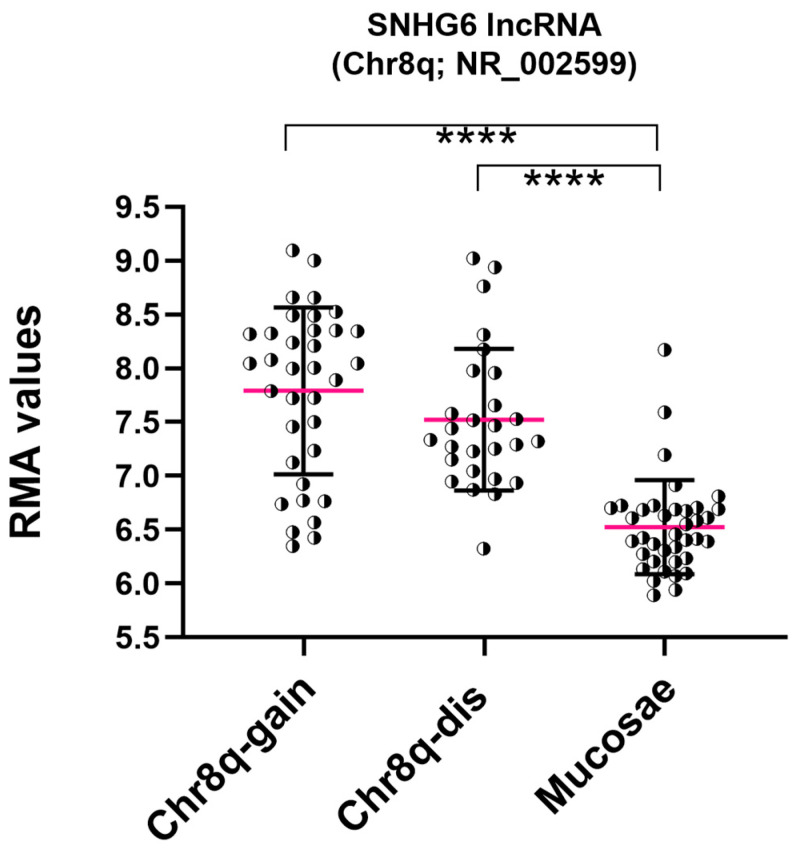
RMA values of SNHG6 in CRC (Chr8q-gain and disomic) and mucosae samples (FC3 > 1.5; FC2 > 1.3) obtained by HTA 2.0. Fuchsia line represents mean value (mean and SD) (**** *p*-value < 0.0001).

Among all six Over-UpT lncRNAs identified in Chr8q, we focused our attention on Over-UpT lncRNA SNHG6 (NR_002599; 727 bp, 4 exons), taking into account the confirmation of this result using TCGA data. RMA values obtained by HTA 2.0 revealed a higher expression of SNHG6 lncRNA in CRC samples with a disomic Chr8q in comparison to Normal Colonic Mucosae (UpT, 1.85-fold). Expression of lncRNA SNHG6 is further increased in CRC samples bearing Chr8q-gain versus disomic Chr8q (Over-UpT, 1.65-fold), as reported in Figure 6.

**Figure 7:**
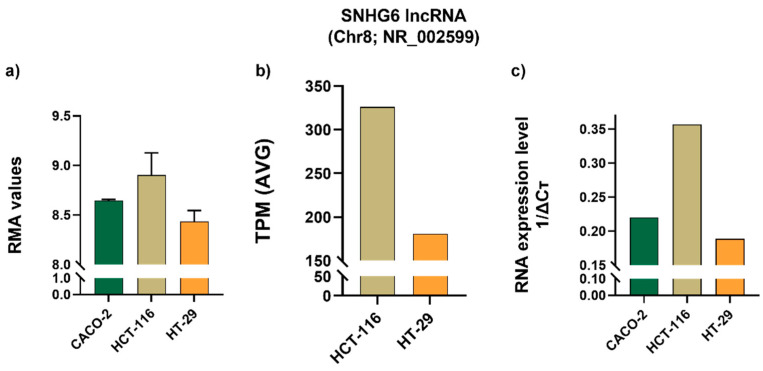
SNHG6 expression in colorectal cancer cell lines: RMA (**a**), TPM (**b**) values and q-RT PCR (**c**).

Furthermore, SNHG6 lncRNA expression has been investigated in CRC cell lines. Genomic analysis by SNP array 6.0 revealed that HCT-116 cell lines are characterized by Chr8q-gain, while CACO-2 cell lines do not carry 8q aberration. HTA (Figure 7a), RNA-seq (Figure 7b) and q-RT PCR (Figure 7c) data confirmed a higher expression of SNHG6 lncRNA, located on chromosome 8q, in the HCT-116 cell line, compared to CACO-2.

### 2.5. miRNAs Interacting with NORAD and SNHG6

**Figure 8:**
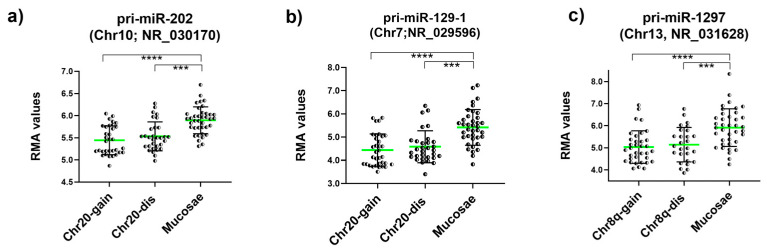
RMA values of pri-miR-202 (NR_030170), (**a**), pri-miR-129-1 (NR_029596), (**b**) and pri-miR-1297 (NR_031628), (**c**) in CRC and mucosae samples obtained by HTA 2.0 (mean and SD). Green line represents mean value (**** *p*-value < 0.0001; *** *p*-value < 0.001).

Prediction tools have allowed us to recognize specific miRNA-Over-UpT lncRNAs’ interactions, underlying the sponging mechanism of Over-UpT lncRNAs. Bioinformatic algorithms have been used to predict the microRNAs (miRNAs) that might be latently sponged by NORAD and SNHG6. Among them, we selected miR-202 and miR-129-1 predicted for NORAD and miR-1297 for SNHG6. The lncRNAs and miRNA sequences downloaded from NCBI (https://www.ncbi.nlm.nih.gov accessed on June 2023) and their interactions are reported in Figure S1A,B. The precursors of these miRNAs (pri-miRNAs) were analyzed in the same cohort of colon cancer and mucosae samples, using HTA 2.0. Interestingly, pri-miR-202 (Chr10), pri-miR-129-1 (Chr7) and pri-miR-1297 (Chr13) are significantly under-expressed in CRC samples compared to mucosae (Figure 8a–c), supporting the hypothesis of a NORAD/SNHG6-mediated sponging mechanism.

**Figure 9:**
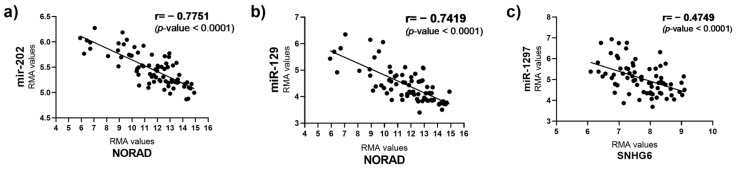
(**a**) Pearson’s correlation between NORAD and miR-202 expression levels in CRC samples; (**b**) Pearson’s correlation between NORAD and miR-129 expression levels in CRC samples; (**c**) Pearson’s correlation between SNHG6 and miR-1297 expression levels in CRC samples.

The correlation between the expression levels of NORAD, pri-mir-202 and pri-mir-129 was analyzed. As shown in Figure 9, pri-mir-202 (Figure 9a) and pri-miR-129 (Figure 9b) levels are inversely proportional at the levels of lncRNA NORAD. A correlation value of −0.7751 and −0.7419 indicates a significant and negative relationship between NORAD and the two pri-miR transcripts. The correlation between the expression levels of SNHG6 and pri-mir-1297 was also analyzed, and it follows the same trend just described, even if at a lower value (r = −0.4749) (Figure 9c).

**Figure 10:**
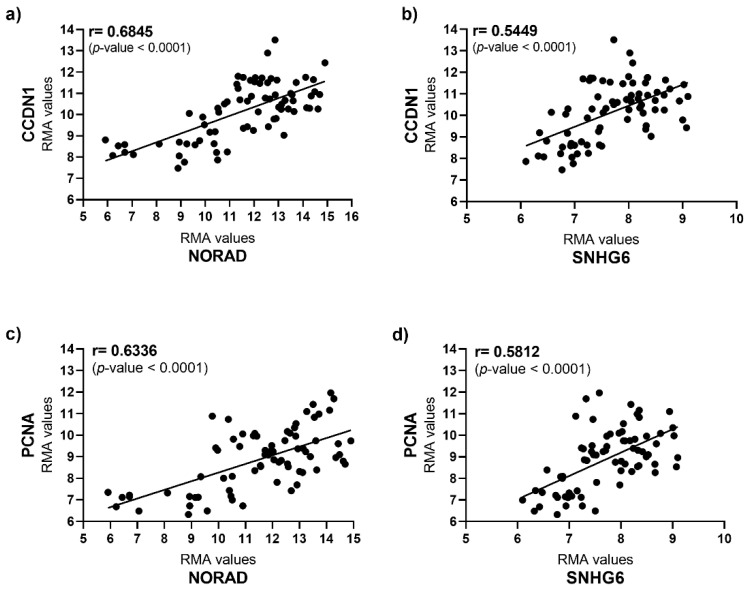
Pearson’s correlation between NORAD (**a**)/SNHG6 (**b**) expression levels and CCD1 in CRC samples. Pearson’s correlation between NORAD (**c**)/SNHG6 (**d**) expression levels and PCNA in CRC samples.

The correlation between Over-UpT lcnRNAs (NORAD and SNHG6) and transcripts coding for protein associated with cell proliferation, such as Cycline D1 (CCND1) and proliferating cell nuclear antigen (PCNA), was also evaluated. As shown in Figure 10a–d, the levels of the lncRNAs NORAD and SNHG6 are directly and significantly correlated with CCND1 and PCNA transcripts, suggesting a strong correlation with cell proliferation.

## 3. Discussion

The cancer heterogeneity requires research with more in-depth molecular characterization, with the aim of identifying new diagnostic and prognostic biomarkers. Each cancer type shows a unique model of aneuploidy, with whole arms or chromosomes altered at different frequencies [5]. The main mechanism linking chromosomal aberrations to a cancer phenotype consists of the so-called gene dosage transcriptional effect, wherein transcript levels of a significant proportion of genes are correlated to gene copy numbers [8,29,30,31,32]. Correlation studies between genomic and transcriptomic profiles in colorectal cancer were already conducted involving coding regions, suggesting that broad copy number gains, specifically in Chr20, Chr8q, Chr13 and Chr7, have a pivotal role to initiate and sustain the CRC. Here, by employing the same comprehensive genome-transcriptome approach, we elucidate the influence of broad gains on the transcription of non-coding regions. Specifically, we selected lncRNAs showing an expression profile compatible with the definition of Over-UpT genes [8,29]. The HTA 2.0 microarrays utilized probes to assess the expression of 22,829 non-coding transcripts across all 24 chromosomes, including X and Y. Our analysis has been specifically conducted on targeted chromosome gains identified in CRC, encompassing the following annotated lncRNAs recognizable on HTA 2.0 with NR_accession number: n = 46 in Chr20, n = 30 in Chr13, n = 42 in Chr8q and 127 in Chr7. Among these, we identified a total of 55 UpT lncRNAs, with 21 of them recognized as Over-UpT lncRNAs. Well-documented scientific evidence is reported for some of them for their involvement in cancer (NORAD, SNHG6, NFS1, SLMO2-ATP5E, RAB5IF, EDEM2, VAPB, RBM39, TPD522, CPN1, SRSF6), and they gave us good confidence in the validity of the model adopted, suggesting the necessity of further analysis to investigate their involvement in the mechanisms supporting CRC pathogenesis. Among the Over-UpT lncRNAs worth being noted are NORAD and SNHG6, located, respectively, on chromosomes 20 and 8. 

Lee et al. investigated NORAD’s role by creating a NORAD-deficient human cancer cell line using genome editing methods [33]. Their findings revealed that cells lacking NORAD showed significant chromosomal instability, marked by increased chromosome loss and gain. Interestingly, reintroducing NORAD expression reversed this instability, highlighting its crucial role in maintaining chromosomal integrity. NORAD contributes to genomic stability by sequestering PUMILIO proteins, belonging to the PUF (PUMILIO-Fem3-binding factor) protein family, which are deeply conserved RNA-binding proteins. The PUF proteins bind with high specificity to the 3′ UTR sequence of their mRNAs’ target via the PUMILIO homology domain, reducing translation and promoting degradation, thus acting as negative regulators of the expression gene [33]. Genes that are crucial for chromosomal stability, mitosis, DNA repair and replication are targeted by PUM proteins. 

Due to its numerous PUM binding sites, NORAD can effectively sequester a significant portion of cellular PUM proteins, thus serving as a negative regulator of PUM activity and preserving genomic stability. Therefore, Lee et al. defined NORAD as a decoy for PUM1/PUM2 [33]. Although NORAD’s involvement in maintaining genomic stability makes it a potential tumor suppressor, its impact on cancer remains intricate and contradictory. NORAD behaves as an oncogene in many human cancers, where it is upregulated. Wang et al. (2018) demonstrated the upregulation of NORAD in the CRC tissues compared to the adjacent normal mucosae [18]. NORAD is implicated in various aspects of carcinogenesis, encompassing proliferation, invasion, metastasis and apoptosis, mediated by multiple mechanisms and signaling pathways, including inducing epithelial-to-mesenchymal transition (EMT) and sponging tumor-associated miRNAs [34]. Interestingly, NORAD could regulate cancer progression by acting as competing endogenous RNAs to modulate miRNA expression and function [35]. Zhang et al. demonstrated that NORAD overexpression promotes proliferation, migration and invasion in CRC cells while inhibiting apoptosis by downregulating miR-202-5p [36]. Expression of lncRNA NORAD has been investigated in our cohort of CRC samples and colon cell lines, and resulted further increased in CRC bearing Chr20-gain versus disomic Chr20, directly related to the cell proliferation markers (CCND1 and PCNA), suggesting that NORAD is sensitive to the gene dosage associated with the increase in the number of copies of the long arm of Chr20. Bioinformatics databases were used to predict NORAD–miRNA interactions. The results showed that NORAD contained a potential binding site for miR-202 and miR-129-1 [36,37]. In our study, the precursors of these miRNAs (pri-miR-202 and pri-miR-129-1) were analyzed in the same cohort of colon cancer and mucosae samples by HTA 2.0 and, interestingly, are significantly under-expressed in CRC samples compared to mucosae. Furthermore, NORAD and pri-miR-202 and pri-miR-129-1 show a negative Pearson’s correlation, suggesting the hypothesis of a NORAD-mediated sponging mechanism. Our data agree with the results proposed by Zhang and suggest that the NORAD potential sponge effect may also occur in the nuclear compartment at the level of primary transcripts (pri-miRNAs). Further systematic investigation is required to thoroughly understand the dual roles of NORAD and to clarify the molecular mechanisms both upstream and downstream of NORAD, in order to gain insight into its potential role in colorectal cancer.

Several studies have reported a higher expression of lncRNA SNHG6 in CRC tissues and demonstrated its correlation to the TNM stage and shorter overall survival [38]. Additionally, its overexpression in CRC cell lines led to increased cell proliferation, migration and invasion, whereas its knockdown results in increased apoptosis [39,40]. Although SNHG6 has been reported to be a potential oncogene in colorectal cancer, its association with chromosomal instability and BCNAs has not yet been investigated. In the present study, the expression of lncRNA SNHG6 has been determined in CRC samples and colon cell lines and resulted further overexpressed in CRC-bearing Chr8q-gain versus disomic Chr8q and directly related to the cell proliferation markers (CCND1 and PCNA). SNHG6 could act as a competing endogenous RNA in human tumors [38]. To investigate whether miRNAs are involved in the mechanism underlying the role of SNHG6, software was used to predict SNHG6–miRNA interactions. The results showed that SNHG6 contained a potential binding site for miR-1297. Their link has already been discussed in hepatocarcinogenesis, where SNHG6 acts as a trigger of hypomethylation at the genomic level via coupling of miR-1297 [41]. The precursors of miR-1297 (pri-miR-1297) have been analyzed in the same CRC and mucosae cohort by HTA 2.0 and, interestingly, it is significantly under-expressed in CRC samples compared to mucosae. Furthermore, SNHG6 and pri-miR-1297 show a negative Pearson’s correlation, supporting the hypothesis of an SNHG6-mediated sponging mechanism.

In summary, we provide evidence that the combination of transcriptional indices provides a tool to select specific candidate lncRNAs involved in colorectal cancer initiation and progression. Experimental characterization of their associated molecules, such as miRNAs or proteins, is needed to confirm the role of identified lncRNAs.

## 4. Materials and Methods

### 4.1. Human Cell Lines

The human colorectal adenocarcinoma HT-29 (ATCCR HTB-38™), CACO-2 (ATCCR HTB-37™) and HCT-116 (ATCCR CCL-247™) cell lines were provided by the American Type Culture Collection (Manassas, VA, USA). 

HT-29 and CACO-2 cell lines were maintained in Dulbecco’s Modified Eagle Medium (DMEM 1X; GIBCO, Cat. No. 31965-023, containing 4.5 g L-1 of D-glucose), supplemented with 10–20% fetal bovine serum (Cat. No. 10270-106; Life Technologies, Carlsbad, CA, USA) and 1% antibiotics (100 unit/mL penicillin and 100 μg/mL streptomycin) (Cat. No. 15140-122; Life Technologies).The base medium for the formulated HCT-116 cell line is McCoy’s 5a Medium Modified, supplemented with fetal bovine serum for a final concentration of 10% and 1% of penicillin–streptomycin. The cell cultures were grown in flasks (25 cm^2^) and incubated at 37 °C in a humidified atmosphere consisting of 5% CO_2_ and 95% air.

### 4.2. Genomic DNA Extraction

Genomic DNA was extracted from each cell line using a Dneasy Blood & Tissue Kit (Cat. No. 69504 and 69506; Qiagen, Milan, Italy), according to the manufacturer’s instructions. The concentration and the quality of the DNA were determined using an ND-1000 spectrophotometer (NanoDrop, Thermo Scientific, Waltham, MA, USA).

### 4.3. Molecular Karyotyping by SNP 6.0 arrays

A genome-wide DNA copy number and an SNP genotyping analysis was performed in three colon cancer cell lines on Affymetrix SNP 6.0 arrays (Affymetrix, Inc., Santa Clara, CA, USA), using 500 ng of input DNA, as previously described [6,42]. Array scanning and data analysis were performed by using the Affymetrix^®^ “GeneChip Command Console” (AGCC) and the “Genotyping Console™” (GTC) version 3.0.1 software. BCNAs, defined as gains or losses involving more than 25% of a chromosomal arm or numerical aberrations involving whole chromosomes, were identified as described by Barresi et al., 2017. Raw and processed data of colon cell lines obtained in SNP 6.0 arrays have been submitted to the public repository, “Gene Expression Omnibus-GEO” (www.ncbi.nlm.nih.gov/geo), and are accesible with the following accession number: GSE267025.

Moreover, data obtained by molecular karyotyping of CRC samples (46 tumoral samples and 26 normal matched mucosae) were deposited in the public repository “Gene Expression Omnibus-GEO” (www.ncbi.nlm.nih.gov/geo accessed on April 2018), and are accessible through GEO with the following accession number: GSE80460 [4]. The cytogenetic results of 439 COAD samples (Affymetrix SNP 6.0) were downloaded from cBioPortal for cancer genomics (https://www.cbioportal.org accessed on September 2019) [43,44]. Only samples matched to TCGA-COAD RNA-Seq data were selected for further analysis (n = 433 samples). 

### 4.4. RNA-Seq Data 

The colon adenocarcinoma (COAD) samples were downloaded from The Genomic Data Commons (GDC) Data Portal (https://portal.gdc.cancer.gov accessed on September 2019) by selecting the RNA-Seq counts data from The Cancer Genome Atlas (TCGA) [27,28]. We analyzed 433 primary tumor COAD samples having both RNA-seq data and cytogenetic results (Affymetrix SNP 6.0 arrays), and 41 mucosal normal samples. All count data were normalized with the “trimmed mean of M-values” method introduced by Robinson and Oshlack. Differential expression of transcripts was analyzed using the R packages edgeR v.3.26.8 and compcodeR v.1.18.1 (accessed on September 2019) [45,46,47]. The *p*-values were adjusted for multiple comparisons using the Benjamini–Hochberg correction [48]. Furthermore, RNA-seq cell lines data were downloaded from a publicly available database (https://cellmodelpassports.sanger.ac.uk accessed on May 2022) as raw counts and converted into FPKM (Fragments Per Kilobase Million) and TPM (Transcripts Per Kilobase Million) data.

### 4.5. Transcriptome Analysis by Human Transcriptome Array 2.0

A transcriptome analysis has been performed using the Human Transcriptome array 2.0 on the local cohort, including 46 tumoral samples and 26 normal matched mucosae, and data have been deposited to the public repository “Gene Expression Omnibus-GEO” (www.ncbi.nlm.nih.gov/geo accessed on September 2018); they are accessible through GEO using the following accession numbers: GSE73360 and GSE84984 [49,50,51]. Data have been elaborated using Affymetrix^®^ Transcriptome Analysis Console^TM^ (TAC) 2.0 Software (Affymetrix UK Ltd., High Wycombe, UK), which performs statistical analysis and identifies differentially expressed genes. A transcript level analysis was performed using the normalization method based on the processing algorithm called robust multiarray average (RMA). Average RMA values have been transformed in linear values, and their ratios (linear fold-changes) have been used in order to estimate differential expression between CRC groups and the normal colon group. Linear fold-changes were calculated in the following way: 2 ^[study group Average RMA − control group Average RMA]^ if study group > control group, or −2 ^[control group Average RMA − study group Average RMA]^ if study group < control group. 

### 4.6. RNA Extraction and Quantitative q-RT PCR

RNA from colorectal cancer cell lines was extracted using an Rneasy Mini Kit (Cat. No. 74104, Qiagen, Milan, Italy) and was quantified using the NanoDrop spectrophotometer. Reverse transcription was performed starting with 1 μg of total RNA, using the High-Capacity RNA to cDNA Kit (Cat. No.4368813, ThermoFisher, Milan, Italy). Primers were designed by the “Primers-BLAST” tool from NCBI (https://www.ncbi.nlm.nih.gov/tools/primer-blast accessed on June 2023); below, in Table 2, are reports of each transcript forward and reverse sequence primer, annealing temperature and fragment size. 

qRT-PCR analysis was performed using a StepOne^TM^ Real-Time PCR System and a Power SYBR™ Green PCR Master Mix (Catalogue number: 4367659) by Applied Biosystems (Applied Biosystems, Foster City, CA, USA), according to the manufacturer’s protocol. Each sample was analyzed in triplicate and the average was normalized to human ACTB expression. Amplification conditions included a cycle at 95 °C for 10 min, followed by 40 cycles at 95 °C for 15 s and 56–61 °C for 1 min. As a negative control, reaction without cDNA was performed (no template control, NTC). The RNA expression level for each sample was calculated using ΔCt values, normalized versus ACTB and reported as 1/ΔCt.

### 4.7. Prediction of Targeting Relationship: lncRNA-miRNA-mRNA

The correlations of lncRNA-miRNA, miRNA-mRNA and lncRNA-mRNA pairs were evaluated using the starBase database (https://starbase.sysu.edu.cn accessed on June 2023), which is an opensource platform for studying the ncRNA interactions from CLIP-seq, degradome-seq and RNA-RNA interactome data [52,53]. We integrated the miRNA–lncRNA and mRNA–miRNA interacting pairs from miRNet (https://www.mirnet.ca accessed on June 2023). Additional databases used for target prediction include miRanda and TargetScan. The selection was made on the basis of different parameters provided by the databases, including affinity of miRNA-lncRNA interaction. In order to improve reliability of the results, we only selected for further analyses of those miRNA–lncRNA pairs that overlapped in both databases.

### 4.8. Statistical Analysis

Statistical analysis was conducted using GraphPad Prism 8 (v.8; GraphPad Software Inc., La Jolla, CA, USA). ANOVA was used to analyze differences between groups. Pearson coefficients were calculated to estimate correlations between data-sets. A *p*-value of less than 0.05 was considered statistically significant. FDR (False Discovery Rate) analyses (Table S1), according to the Benjamini–Hoechberg correction [48], were performed using the Affymetrix software^®^ Transcriptome Analysis Console 2.0 (Affymetrix, Inc., Santa Clara, CA, USA).

## 5. Conclusions

These results led us to obtain a list of upregulated lncRNAs in colorectal cancer. Only a subgroup of UpT lncRNAs are sensitive to BCNA dosage effects.This subset, called Over-UpT lncRNAs, are upregulated in CRC and further overexpressed in colon tumors bearing specific chromosomal aberrations. In particular, Over-UpT lncRNAs NORAD and SNHG6 are significantly associated with the increase in the number of copies of chromosomes 20 and 8q, where NORAD and SNHG6 are, respectively, located. The candidate lncRNAs associated with BCNA need to be further investigated to evaluate their role in the development and progression of colorectal cancer. 

In conclusion, further studies are necessary to evaluate a possible use of lncRNA as a therapeutic target based on transcriptional silencing. Antisense oligonucleotides (ASOs) can be employed either to induce lncRNA degradation or prevent its interaction with other regulatory molecules [54]. An alternative approach to target lncRNA expression is based on RNA interference, through lncRNA-specific siRNAs [55,56]. Depletion of specific lncRNAs or the gain of specific correlated miRNAs could repress proliferation and metastasis, indicating a positive correlation between lncRNA and mRNA targeted by miRNA sponging.

## Figures and Tables

**Table 1:** UpT LncRNAs in CRC.

UpT LncRNAs in CRC			
Chr7 (n = 22)	Chr8q (n = 9)	Chr13 (n = 7)	Chr20 (n = 17)
Gene Symbol	Gene Symbol	Gene Symbol	Gene Symbol
RNF216P1 (NR_023385)	SNHG6 (NR_002599)	PSPC1 (NR_044998)	NSFL1C (NR_038164)
PHF14 (NR_033435)	TMEM70 (NR_033334)	RPL21P28 (NR_026911)	NOP56 (NR_027700)
RPS2P32 (NR_026676)	ZFAND1 (NR_033193)	COG6 (NR_026745)	MAVS (NR_037921)
GGCT (NR_037669)	NACA4P (NR_002182)	ST13P4 (NR_002183)	NDUFAF5 (NR_029377)
HUS1 (NR_037917)	ENY2 (NR_036471)	ALG11 (NR_036571)	EDEM2 (NR_026728)
FKBP9P1 (NR_027339)	TATDN1 (NR_027427)	UBAC2 (NR_026644)	CPNE1 (NR_037188)
CRCP (NR_024548)	PVT1 (NR_003367)	TFDP1 (NR_026580)	NFS1 (NR_037570)
GTF2IP4 (NR_003580)	SHARPIN (NR_038270)		RBM39 (NR_040722)
BCL7B (NR_036682)	ZNF252P (NR_023392)		NORAD (NR_027451)
BUD23 (NR_037776)			RAB5IF (NR_026562)
NSUN5P1 (NR_033322)			NDRG3 (NR_038370)
PMS2P3 (NR_028059)			SRSF6 (NR_034009)
SLC25A13 (NR_027662)			ZFAS1 (NR_003604)
TAF6 (NR_033792)			PEDS1 (NR_027889)
PMS2P1 (NR_003613)			VAPB (NR_036633)
LOC100630923 (NR_038967)			SLMO2-ATP5E (NR_037930)
FAM185A (NR_026879)			TPD52L2 (NR_045090)
BCAP29 (NR_027830)			
CBLL1 (NR_024199)			
POT1 (NR_003102)			
LINC01000 (NR_024368)			
TNPO3 (NR_034053)			

**Table 2:** Sequence primers.

Targets	Forward Primer	Reverse Primer	Annealing Temperature	Fragment Size
ACTB NM_001101.5	AGAGAGGCATCCTCACCCTG	ATAGCACAGCCTGGATAGCAA	59/57	240 bp
SNHG6 NR_002599.2	GTTAGTCATGCCGGTGTGGT	AATACATGCCGCGTGATCCT	57/55	171 bp
NORAD NR_002599.2	CAGACTTTGCTGTCGGAAGA	ACACAGGCCTTCCATAAACG	55/55	113 bp

## Data Availability

Human SNP 6.0 and transcriptomic array data are available on GEO datasets (GEO: GSE80460, GSE267025, GSE73360, GSE84984).

## Supplementary Materials

The supporting information can be downloaded at: https://www.mdpi.com/article/10.3390/ijms25105538/s1.
