# Supplementary material for: Gain-Type Aneuploidies Influence the Burden of Selective Long Non-Coding Transcripts in Colorectal Cancer
Source: Int J Mol Sci. 2024 May 19;25(10):5538. doi: 10.3390/ijms25105538 (PMC11122260; doi:10.3390/ijms25105538)
Supplement: Supplementary file 1 [file ijms-25-05538-s001.zip › Figure S1.pdf]

**Figure S1A,B.** Nucleotide sequences of NORAD (NR_027451), SNHG6 (NR_002599) lncRNAs and corresponding miRNAs binding sites based on the use of prediction tools are reported.

**Figure S1A.** Nucleotide sequences of NORAD (NR_027451) and corresponding miRNAs binding sites (miR-202 and miR-129-1) are highlighted respectively in yellow, green and fuchsia. Bold nucleotides indicate the region involved in the matched lncRNA-miRNA. Target-directed microRNA degradation (TDMD Score) is indicated.

## NORAD

**ACCESSION** NR_027451,5378 bp
**DEFINITION** Homo sapiens non-coding RNA activated by DNA damage (NORAD, long non-coding RNA.
**CHROMOSOME** 20q11.23

ORIGIN

3' → 5'

1 agttccggtc cggcagagat cgcggagaga cgcagaacgc agcccgctcc tccagggccc
61 tccaggccct ccggccccgg gccggcgggt gaactggggg gccccgggac aggccgagcc
121 ctctgccctg cagataacgg aggcctctgc tgtggctgcc cactggctgt gcccgcccac
181 tggctgtgcc cagaccttga agccgcagcg aacctctctt tcccacccca cctcggtgac
241 taatggcggc cgtggcgtct cccagcccgg accccgccgg cacccgggtc tcccgaccca
301 agcctcgacg aaaccccccgc agagccgccg ggacgcagcg cctttgggcg gcgctgggcg
361 tggtgggccg ggaagtatgg cggcagctcg aacgccgcgc ggcggaggcc attaaggcgt
421 ggacggcccg ggaaggcggc ctagggacgc aagcaggctc ggccgcctct ttaggccacg
481 gagccgcgca gatccggttc ccgggtgacc actctgtcgc cattgggcga gacctaccta
541 gtcctgacga caacggacaa aggccttaag gggcctggaa ggtgagcgaa gtcccgaacg
601 acgacgggtg gaacggttag cggccatcgg gcggttggtc ttcattctac cagactttgc
661 tgtcggaaga gagaaatggt agaatgacag gccacgtttg gcccgttgga aatgcccacc
721 accctctggg aagatttact ggccgtttat ggaaggcctg tgtatataat atgaaaaagc
781 tgctctcaac tccaccccaa ccttttaata gaaaacattt gtcacatcta gcccttctag
841 atggaaagag gttgccgacg tatgataaaa tagagttaga aagttacaca tcttgtaaat
901 tctcatttgt ttaaaagaaa tcatagaaaa tacatgtctt ctggagatga cttttggaaa
961 tggagttgtt aagacggcct ctggaagcga tacgtccacg tttgttaagt gggttagatg
1021 acatggagct ggaagacctg agaaggaaga gaagaaggtt ctatgctaga ctggtcatat
1081 ttagaagaca ttttcatatt ctatccattg ttttgtgtgc attttattcc tcactactgt
1141 gtatatagtt gacaatgcta agctttttttg aaatgtctct tctttttaga tgttctgaag
1201 tgcctgatat gttaaaatta gaggtagcaa aatcacattt tgtaaatacc tttttgttac
1261 aattcatagg aaatattttt gggggggaat ggccaaatca cctgttgagt aatactcatt
1321 gtgtttgtgc agtggttcag gggaggagag aggaggggga ggtgcagaga gctctatgcc
1381 atcctgttta cagcgaggca agatgaatca ttatgtctgt gcattttgtt ttacttatct
1441 gtgtatatag tgtacataaa ggacagacga gtcctaattg acaacatcta gtctttctgg
1501 atgttaaaga ggttgccagt gtatgacaaa agtagagtta gtaaactaat atattttgta
1561 cattttgttt tacaagtcct aggaaagatt gtcttctgaa aatttgatgt cttctgggtt
1621 gatggagatg ggaagggttc taggccagaa tgttcacatt tggaagactc tttcaaatta
1681 taactgttgt tacatgtttg cagtttattc aagactgctg tatacatagt agacaaatta
1741 actccttact tgaaacatct agtctatcta gatgtttaga agtgcccgat gtatgttaaa
1801 tgtataggta gtaaaatacc actttgtaaa tatcttttttg ctaaaattca taggaaatgc
1861 ttttggaaat tgaattgtga agccaccttt gtgaacagta tagtaatgtc tatacttgtt
1921 caatagttta gaggaggtag gagggaagaa attgcaaaag gtaatattac tagtgtgttc
1981 atacttggac attttcagac accatttttc tatatgtttt gtgcattttg ttttgctctg
2041 tatatagtat atataatgga caaatagtcc taatttttca acatctagtc tctagatgtt
2101 aaagaggttg ccagtgtatg acaaaggagt aaaattagca tattttgtac actttgtgtt
2161 gaaattcgta ggaaaacttg tcttct**gtaa agacttttgc ataggaa**ttt gtttgaccat
2221 ctctaagcat tacacgtgcc tgtacttgtc cactggattg aaggcagaga aggaagggag
2281 gagggaatga ttcaaggcca aaatggccac atttagaaga tacctcagat gataaccatt
2341 gttatgtgtg tgcaatttta tttaacagtg ctgtgtatgt ggtggacaag ttatatgaaa
2401 tatctagtct ttctagatat ttggaagtgc ttgatgtatt taaaagtggt agtagaataa

2461 cactttgtaa atagctttta aaaactgatg ggaaatgctg tttggaagtg gaattgttga
2521 accacctggg aggtgggagg gaagaaattg caaatggtgt tttgccattg tttattagaa
2581 aatttcagct taatccattg tgtatatgtt acatgcattt catttaactt tgctatactg
2641 tatatattgt atatataacg gacaaattag tcccgatttt ataatatcta gtctctagat
2701 attaaagagg ttgccaatgt atgacagaag tagagttagt aaactaacac attttgtaca
2761 ctttgttaaa atttgtagaa aggctgtctt ctgaaaagga cttttggaag tgagataaca
2821 tcagctctaa gtgacacgtg cctatatcca tcaggttggt ggtggagagg agttggaagg
2881 aatgaagggt tctagaccag aatgttcgta tttagaagac actatcagat ataaccattg
2941 ttacatgtgt gtagtttatt caaccctact gtgtatatag cggacaaact taagtcctta
3001 tttgaaacat ctagtctttc tagatgttta gaagtgcaca aagtatgtta aaagtagagg
3061 tagtaaataa cacattttgt agctatcctt ttgatatgaa atattgtctt ggaaattgat
3121 caattctctg agcagtaccc attttgatat ttgtgctggt tcaggggaa ggag**gagcac**
3181 **aaagtgcaaa** **gggctt**tcta ccagtgtcca gtgtgtttat gaggaggcac attgaccatt
3241 gtcccttatg tctgcatttt catttactgt gctgtgtata tagtgtatat aagcggacat
3301 aggagtccta atttacgtct agtcgatgtt aaaaaggttg ccagtatatg acaaaagtag
3361 aattagtaaa ctactacatt gagtacactt tgtgttaaaa ttcataggga agacttctta
3421 aaaacaagtg aaattgttaa aacccccccct aagcattaca gatggcttat agctgtccac
3481 ggggttggta gaggtgggaa agggaagggt tctaggccag aatgttccta tttagaagac
3541 actcaaatta cagtctgtgt tatgtatgta taccatttat tcaatgctac tgtgtatata
3601 atggaaaact taagtccagt ttgaaacatc tagtctttct aggtgtttaa aagtgtacaa
3661 cggcctgtcg cagtggcgca tgcctgtaat cccagcactt tgggaggccg aggcaggcgg
3721 atcacgaggt caagagatca ggaccatctt ggccaacatg gtgaaacccc atctttacta
3781 aaaatacaaa aattagctgg tcgtggtggt gcccacctgt agccccagtt actcgagagg
3841 ctgaggcagg agaatcgctt gaacttggga ggcggaagtt gcagtgagcc aagatcgcac
3901 cactgcactc cagcctggcg acagagcgag gctccgtttc aaaaaaaaaa gtgcacaatg
3961 taggttaaca gtagagggct taagtaacac ccctctaagc atttgttttc agtacttcct
4021 aggagtggtt gcatttggga atggaattgt taaaacttga tgcttaggag cgaatgcaga
4081 ctattcattg ggtgtttggg gtgggggaag ggggggtggg cagaggaggt atgcagggag
4141 aggggttctg tgctcctgag attagttcag atggtctaac cattgttcta tatgtgcatt
4201 ttagttaata ttgtgtatta aaggataagt cttaatgctc aaagtatgtt aaaaatagat
4261 gtagtaaatc agtccctttg tgaatgtcct tttgttagtt tttaggaagg cctgtcctct
4321 gggagtgacc tttattagtc caccccttgg agctagacat cctgtactta gtcacgggga
4381 tggtggaaga gggagaaga**g** **gaagggtgaa** **gggaagggct** **c**tttgctagt atctccatat
4441 ctagacgatg gttttagatg ataaccacag gtctacaaga gcgtttttag taaagtgcct
4501 gtgttcattg tggacaaagt tattattttg caacatctaa gctttacgaa tggggtgaca
4561 acttatgata aaaactagag ctagtgaatt agcctatttg taaatacctt tgttataatt
4621 gataggatac atcttggaca tggaattgtt aagccacctc tgagcagtgt atgtcaggac
4681 ttgttcatta ggttggcagc agaggggcag aaggaattat acaggtagag atgtatgcag
4741 atgtgtccat atatgtccat atttacattt tgatagccat tgatgtatgc atctcttggc
4801 tgtactataa gaacacatta attcaatgga aatacacttt gctaatattt taatggtata
4861 gatctgctaa tgaattctct taaaaacata ctgtattctg ttgctgtgtg tttcatttta
4921 aattgagcat taagggaatg cagcatttaa atcagaactc tgccaatgct tttatctaga
4981 ggcgtgttgc catttttgtc ttatatgaaa tttctgtccc aagaaaggca ggattacatc
5041 tttttttttt tttttagcag tttgagttgg tgtagtgtat tcttggttat cagaatactc
5101 atatagcttt gggattttga attggtaaat attcatgatg tgtgaaaaat catgatacat
5161 actgtacagt ctcagtccca taaaattgga tgttgtgcct acacacagga tctagaagaa
5221 tatgtcaaac tataaactgc ttgtgattgt gaatgacttt gttctttgct tgtgtttttc
5281 aatttcctat aatgcacata ctaactttta aaaaataaag gttattttaa aagcctgtat
5341 taaaaaaaaa aaaaaaaaaa aaaaaaaaaa aaaaaaaa

//

**miR-202** (TDMDScore 1.0643)
**ACCESSION** NR_030170,110 bp
**DEFINITION** Homo sapiens microRNA 202 (MIR202), microRNA.
**CHROMOSOME** 10q26.3

ORIGIN

5’ 3’

1 cgcctcagag ccgcccgccg ttcctttt**ttc** **ctatgcatat** **acttctttg**a ggatctggcc
61 taaagaggta tagggcatgg gaaaacgggg cggtcgggtc ctccccagcg

//

**miR-129-1** (TDMDScore 0.2487)
**ACCESSION** NR_029596, 72 bp
**DEFINITION** Homo sapiens microRNA 129-1 (MIR129-1), microRNA.
**CHROMOSOME** 7q32.1

ORIGIN

5’ 3’

1 ggatcttttt gcggtctggg cttgctgttc ctctcaacag tagtcagg**aa** **gcccttaccc**
61 **caaaaagtat** ct
//

**miR-129-1** (TDMDScore 0.4601)
**ACCESSION** NR_029596, 72 bp
**DEFINITION** Homo sapiens microRNA 129-1 (MIR129-1), microRNA.
**CHROMOSOME** 7q32.1

ORIGIN

5’ 3’

1 ggatcttttt gcggtctggg cttgctgttc ctctcaacag tagtcagg**aa** **gcccttaccc**
61 **caaaaagtat** ct
//

**Figure S1B.** Nucleotide sequences of SNHG6 (NR_002599) and corresponding miRNAs binding sites (miR-1297) are highlighted respectively in blue. Bold nucleotides indicate the region involved in the matched lncRNA-miRNA.

## SNHG6

**ACCESSION** NR_002599,727 bp.
**DEFINITION** Homo sapiens small nucleolar RNA host gene 6 (SNHG6), transcript variant 1, long non-coding RNA.
**CHROMOSOME** 8q13.1

ORIGIN

3' → 5'

1 ctttcccgcg cgaccggcga gggaggaaga agcgcgaaga gccgttagtc atgccggtgt
61 ggtggcggcg gcggagactg cgggcccgta gctgggctct gcgaggtgca agaaagcctt
121 tgaggtgaag gtgtatgaaa gtcatcataa cagatgtttt ccaaaaactt gtagaaggtt
181 gtgaaaaaac tactaggatc acgcggcatg tattgagcat ataggttgct gtagatgaat
241 gttcttagct gtcatgttta aaaatacttc tgcttcgtta cctcaagtgt ggcatgcagc
301 attttggaag gaaaattgaa gacgtgttca agaaaacatg aacagaagca aatgatgaaa
361 a**tgagcattt** **tacttgat**gt tgataacatc acaataaatt atggagaaaa atacatattt
421 ggctaacttt taattgctga acaataaagt gttttctttt aaatcaactc taaatagctc
481 cattctcata gtcactagtc agacctgttt tgaacatatt cgaaagatta taatcttgtc
541 aataattagc ttatttatgg gtggtgattc tcattgaggc tgacagctgg ggagacattg
601 cttgtacctc taggttccct gtctggcttc cccttcagag cctgctgttg taccaggtgg
661 ttgaatctta aaactcttta ataccaaata gcaatcaaat tccccttac aaaaaaaaaa
721 aaaaaaa
//

## miR-1297 (TDMDScore 0.1765)

**ACCESSION** NR_031628, 77 bp.
**DEFINITION** Homo sapiens microRNA 1297 (MIR1297), microRNA.
**CHROMOSOME** 13q14.3

ORIGIN

5' ← 3'

1 tgtttatctc tagggttgat ctattagaat tacttatctg agccaaagta a**ttcaagtaa**
61 **ttcaggtg**ta gtgaaac
//
