# Supplementary material for: Gain-Type Aneuploidies Influence the Burden of Selective Long Non-Coding Transcripts in Colorectal Cancer
Source: Int J Mol Sci. 2024 May 19;25(10):5538. doi: 10.3390/ijms25105538 (PMC11122260; doi:10.3390/ijms25105538)
Supplement: Supplementary file 1 [file ijms-25-05538-s001.zip › Table S1 Rev.pdf]

Table S1. List of annotated UpT lncRNAs (selected by FC3 and FC4 > 1.5) in Chr7, Chr8q, Chr13 and Chr20, recognizable on HTA 2.0 with Transcript Cluster (TC) corresponding to NR accession number. Among them, in green Over-UpT lncRNAs (FC3 > 1.5 and FC2 >1.3). The lncRNAs are shown according to their chromosomal location from p telomere to q telomere. The fold change values (FC3, FC4, FC2), FDR *p*-value and DNA strand orientation for each transcript cluster are also reported.

LncRNAs UpT (n=22) in Chr7
(Among them, Over-UpT n=4, highlighted in green)

Transcript Cluster in HTA 2.0	Symbol (NR_accession number)	Genomic position	Strand	FDR p-value (All Conditions)	FC3 Fold Change (linear) (Chr7-dis vs mucosae)	FC4 Fold Change (linear) (Chr7-gain vs mucosae)	FC2 Fold Change (linear) (Chr7-gain vs Chr7-dis)
TC07002119.hg.1	**RNF216P1** (NR_023385)	5013616-5037800	+	5.86E-07	1.75	1.86	1.06
TC07002148.hg.1	**PHF14** (NR_033435)	11013499-11209250	+	2.06E-07	4.36	5.76	1.32
TC07002199.hg.1	**RPS2P32** (NR_026676)	23530007-23531031	+	5.19E-07	2.31	2.36	1.02
TC07002868.hg.1	**GGCT** (NR_037669)	30536237-30544457	-	1.17E-07	8.72	8.69	-1
TC07002933.hg.1	**HUS1** (NR_037917)	48002885-48019222	-	0.034994	1.59	2.36	1.48
TC07002957.hg.1	**FKBP9P1** (NR_027339)	55748767-55780945	-	0.000003	3.02	3.16	1.05
TC07002383.hg.1	**CRCP** (NR_024548)	65579805-65619553	+	0.000005	3.42	4.19	1.23
TC07002398.hg.1	**GTF2IP4** (NR_003580)	72569012-72621336	+	0.000003	9.33	6.26	-1.49
TC07003017.hg.1	**BCL7B** (NR_036682)	72950683-72971602	-	0.000625	2.7	2.01	-1.34
TC07002401.hg.1	**BUD23** (NR_037776)	73097898-73112542	+	3.84E-08	5.07	5.12	1.01
TC07002414.hg.1	**NSUN5P1** (NR_033322)	75039624-75046065	+	0.000002	1.85	1.87	1.01
TC07003030.hg.1	**PMS2P3** (NR_028059)	75137069-75157453	-	0.0013	1.71	1.86	1.09
TC07003070.hg.1	**SLC25A13** (NR_027662)	95749532-95951459	-	0.000408	1.91	2.78	1.45
TC07003080.hg.1	**TAF6** (NR_033792)	99704693-99716995	-	0.000006	2.49	1.93	-1.29
TC07003082.hg.1	**PMS2P1** (NR_003613)	99918263-99933930	-	0.000309	1.95	1.86	-1.05
TC07002502.hg.1	**LOC100630923** (NR_038967)	102004308-102067129	+	0.000013	1.52	1.63	1.07
TC07002503.hg.1	**FAM185A** (NR_026879)	102389399-102449672	+	0.000003	1.65	2.38	1.44
TC07002518.hg.1	**BCAP29** (NR_027830)	107220422-107263762	+	0.000056	2.63	1.66	-1.59

TC07002520.hg.1	**CBLL1** (NR_024199)	107384279-107402083	+	0.000012	5.23	4.16	-1.26
TC07003148.hg.1	**POT1** (NR_003102)	124462440-124570037	-	0.000008	1.54	1.94	1.26
TC07002575.hg.1	**LINC01000** (NR_024368)	128281295-128301052	+	0.000042	3.39	2.12	-1.6
TC07003162.hg.1	**TNPO3** (NR_034053)	128594234-128695227	-	0.00002	5.67	5.17	-1.1

LncRNAs UpT (n=9) in Chr8q

(Among them, Over-UpT n=4, highlighted in green)

Transcript Cluster in HTA 2.0	Symbol (NR_accession number)	Genomic position	Strand	FDR p-value (All Conditions)	FC3 Fold Change (linear) (Chr8q-dis vs mucosae)	FC4 Fold Change (linear) (Chr8q-gain vs mucosae)	FC2 Fold Change (linear) (Chr8q-gain vs Chr8q-dis)
TC08002368.hg.1	**SNHG6** (NR_002599)	67833919-67838633	-	3.19E-08	1.85	3.05	1.65
TC08001978.hg.1	**TMEM70** (NR_033334)	74888377-74895018	+	0.000002	2.16	2.2	1.02
TC08002403.hg.1	**ZFAND1** (NR_033193)	82613566-82633539	-	1.39E-07	3.54	6.18	1.74
TC08002043.hg.1	**NACA4P** (NR_002182)	102381121-102381823	+	0.000491	1.76	1.52	-1.16
TC08002065.hg.1	**ENY2** (NR_036471)	110346552-110358189	+	9.64E-08	4.24	7.73	1.83
TC08002509.hg.1	**TATDN1** (NR_027427)	125500735-125551329	-	2.83E-07	2.4	3.21	1.34
TC08002099.hg.1	**PVT1** (NR_003367)	128806779-129113503	+	0.000004	1.71	1.88	1.1
TC08002572.hg.1	**SHARPIN** (NR_038270)	145153540-145159138	-	0.000023	1.72	2.01	1.16
TC08002577.hg.1	**ZNF252P** (NR_023392)	146198975-146228285	-	0.000076	1.56	1.68	1.07

LncRNAs UpT (n=7) in Chr13

(Among them, Over-UpT n=3, highlighted in green)

Transcript Cluster in HTA 2.0	Symbol (NR_accession number)	Genomic position	Strand	FDR p-value (All Conditions)	FC3 Fold Change (linear) (Chr13-dis vs mucosae)	FC4 Fold Change (linear) (Chr13-gain vs mucosae)	FC2 Fold Change (linear) (Chr13-gain vs Chr13-dis)
TC13001341.hg.1	**PSPC1** (NR_044998)	20248892-20357159	-	1.28E-07	2.56	2.65	1.04
TC13000978.hg.1	**RPL21P28** (NR_026911)	27825693-27830699	+	4.26E-07	6.34	10.97	1.73
TC13001031.hg.1	**COG6** (NR_026745)	40229764-40326765	+	0.000001	3.02	3.43	1.13
TC13001089.hg.1	**ST13P4** (NR_002183)	50746154-50747751	+	0.000148	1.67	1.58	-1.06
TC13001098.hg.1	**ALG11** (NR_036571)	52586523-52603780	+	6.64E-07	1.86	2.26	1.21
TC13001250.hg.1	**UBAC2** (NR_026644)	99853163-100038753	+	0.000001	3.38	6.57	1.94

TC13001318.hg.1	**TFDP1** (NR_026580)	114239003-114295788	+	6.48E-08	5.09	9.83	1.93

LncRNAs UpT (n=17) in Chr20

(Among them, Over-UpT n=10, highlighted in green)

**Transcript Cluster in HTA 2.0**	**Symbol (NR_accession number)**	**Genomic position**	**Strand**	**FDR p-value (All Conditions)**	**FC3 Fold Change (linear) (Chr20-dis vs mucosae)**	**FC4 Fold Change (linear) (Chr20-gain vs mucosae)**	**FC2 Fold Change (linear) (Chr20-gain vs Chr20-dis)**
TC20001376.hg.1	**NSFL1C** (NR_038164)	1422807-1448337	-	0.000219	2.79	3	1.07
TC20001062.hg.1	**NOP56** (NR_027700)	2633178-2639039	+	3.10E-10	7.67	7.91	1.03
TC20001069.hg.1	**MAVS** (NR_037921)	3827446-3856770	+	0.003129	2.04	2.36	1.16
TC20001111.hg.1	**NDUFAF5** (NR_029377)	13765672-13799067	+	0.000022	1.87	1.98	1.06
TC20001553.hg.1	**EDEM2** (NR_026728)	33703160-33735161	-	0.000004	1.62	2.29	1.41
TC20001558.hg.1	**CPNE1** (NR_037188)	34213953-34252859	-	2.30E-10	4.37	13.51	3.1
TC20001559.hg.1	**NFS1** (NR_037570)	34256610-34287287	-	4.26E-07	2.06	3.46	1.68
TC20001560.hg.1	**RBM39** (NR_040722)	34291531-34330258	-	1.70E-07	2.17	4.28	1.97
TC20000810.hg.1	**NORAD** (NR_027451)	34633540-34638882	-	1.04E-07	13.34	39.74	2.98
TC20001204.hg.1	**RAB5IF** (NR_026562)	35234137-35240960	+	0.000001	2.54	7.16	2.82
TC20001565.hg.1	**NDRG3** (NR_038370)	35280169-35374541	-	0.000006	5.58	6.35	1.14
TC20001227.hg.1	**SRSF6** (NR_034009)	42086504-42092244	+	1.17E-08	3.65	5.59	1.53
TC20001267.hg.1	**ZFAS1** (NR_003604)	47894715-47905797	+	4.44E-10	1.95	2.45	1.25
TC20001629.hg.1	**PEDS1** (NR_027889)	48740274-48770335	-	0.000004	2.8	3.59	1.28
TC20001316.hg.1	**VAPB** (NR_036633)	56964175-57026156	+	6.85E-07	2.23	3.57	1.6
TC20001681.hg.1	**SLMO2-ATP5E** (NR_037930)	57603733-57617901	-	0.000009	3.76	10.67	2.84
TC20001360.hg.1	**TPD52L2** (NR_045090)	62496581-62522898	+	1.69E-07	3.28	5.13	1.56
